# Metagenomic analysis reveals distinct changes in the gut microbiome of obese Chinese children

**DOI:** 10.1186/s12864-023-09805-4

**Published:** 2023-11-29

**Authors:** Ping Li, Jiyang Jiang, Yifei Li, Yue Lan, Fan Yang, Jiao Wang, Yuxin Xie, Fei Xiong, Jinhui Wu, Hanmin Liu, Zhenxin Fan

**Affiliations:** 1 Key Laboratory of Birth Defects and Related Diseases of Women and Children of MOE, Department of Pediatrics, NHC Key Laboratory of Chronobiology, West China Second University Hospital, Sichuan University, Chengdu, 610041 Sichuan China; 2 Key Laboratory of Bioresources and Ecoenvironment (Ministry of Education), College of Life Sciences, Sichuan University, Chengdu, 610065 Sichuan China

**Keywords:** Metagenome, Gut microbiome, Childhood obesity

## Abstract

**Background:**

The prevalence of obese children in China is increasing, which poses a great challenge to public health. Gut microbes play an important role in human gut health, and changes in gut status are closely related to obesity. However, how gut microbes contribute to obesity in children remains unclear. In our study, we performed shotgun metagenomic sequencing of feces from 23 obese children, 8 overweight children and 22 control children in Chengdu, Sichuan, China.

**Results:**

We observed a distinct difference in the gut microbiome of obese children and that of controls. Compared with the controls, bacterial pathogen *Campylobacter rectu*s was significantly more abundant in obese children. In addition, functional annotation of microbial genes revealed that there might be gut inflammation in obese children. The guts of overweight children might belong to the transition state between obese and control children due to a gradient in relative abundance of differentially abundant species. Finally, we compared the gut metagenomes of obese Chinese children and obese Mexican children and found that *Trichuris trichiura* was significantly more abundant in the guts of obese Mexican children.

**Conclusions:**

Our results contribute to understanding the changes in the species and function of intestinal microbes in obese Chinese children.

**Supplementary Information:**

The online version contains supplementary material available at 10.1186/s12864-023-09805-4.

## Background

Obesity has become a serious public health problem globally [1]. As the largest developing country and the second largest economy, China has the world’s largest number of people with obesity or overweight. Based on the newly released 2020 Report on Chinese Residents Chronic Diseases and Nutrition, approximately 50% of adults and 20% of school-age children are overweight or obese in China. Recent research has projected that by 2030, approximately 65.3% of adults and 31.8% of school-age children and adolescents in China could become overweight or obese if no effective interventions are implemented [2]. Childhood is a critical period for growth and development. Now, more than ever before, it has become clear that obese children are prone to becoming obese adults, with higher chances of developing severe comorbidities, such as dyslipidemia, metabolic syndromes, cardiovascular diseases, and type 2 diabetes [3, 4]. Thus, the precise and effective prevention and treatment of childhood obesity as early as possible has significant socioeconomic benefits and is also an important starting point for China’s national population health strategy.

Childhood obesity is a multifactorial disease that can be linked to suboptimal macronutrient composition in the diet, together with insufficient physical activity. At present, an increasing number of studies have shown that the gut microbiome is closely related to obesity, and gut microbes can affect human metabolism by producing metabolites [5–7].

Previous studies have shown that the composition of gut microbes varies from population to population. For example, the abundance of *Bacteroides* was reduced in obese Chinese adults [8], while in obese European adults, *Bacteroides* belonging to the inflammatory enterotype had an increased abundance [9]. Asian-Pacific Islanders possess significantly less Odoribacteriaceae and *Odoribacter* than both Hispanics and Caucasians, which also indicated that gut microbes varies from population to population [10]. Therefore, it is necessary to conduct targeted research on obese children in China since there are few gut metagenomic studies on obese children in China. In addition, many studies were limited to the application of 16S rRNA sequencing technology; while metagenomic sequencing can be used to provide a more comprehensive and in-depth understanding of the gut microbiome and carry out more functional annotation of microbial genes.

In this study, we performed fecal metagenomic sequencing on 23 obese children, 22 controls, and 8 overweight children to analyze the differences in their gut microbiomes. In addition, we compared the difference in the gut microbiome between obese children from China and 10 obese children from Mexico. This study revealed the characteristics of gut microbes in obese children in Chengdu, Sichuan, China.

## Results

### Clinical parameters and sequencing data

A total of 23 obese children (9 males/14 females), 8 overweight children (4 males/4 females), and 22 control children (10 males/12 females) were enrolled in the study from August 2021 to April 2022. The basic characteristics of the participants are presented in Table 1 and Table S1 (Additional file 1). The average months of age of the controls, overweight and obese children was 68.4 ± 7.2, 106.1 ± 42.4 and 108.9 ± 36.9 respectively. We compared the gut microbiome of children with different ages based on the principal coordinate analysis (PCoA) plot, which showed that samples could not be separated by age (Fig. S1a-l; PERMANOVA test of Bray–Curtis dissimilarity; *p* > 0.05). The average BMI of controls was 14.2 ± 1.1 (kg/m^2^), while that of the obese children and overweight children was 23.8 ± 3.3 (kg/m^2^) and 20.3 ± 2.2 (kg/m^2^), respectively.

**Table 1:** The basic characteristics of Chinese participants

	Controls	Overweight	Obese	*p value*
Number of children, n (%)	22 (41.5)	8 (15.1)	23 (43.4)	-
Male, n (%)	10 (45.5)	4 (50.0)	9 (39.1)	> 0.05^a^
Months of age (m) ($\overline{X }$±SD)	68.4 ± 7.2	106.1 ± 42.4	108.9 ± 36.9	1.013e-04^b^
BMI (kg.m^−2^) ($\overline{X }$±SD)	14.2 ± 1.1	20.3 ± 2.3	23.8 ± 3.3	1.802e-09^b^

^a^Refer to the Fisher's exact test

^b^Refer to the Kruskal–Wallis test

We obtained 54,319,772 ± 16,935,752 raw reads by Illumina NovaSeq 6000. The Q20 was 97.2% ± 0.5%, and the Q30 was 92.1% ± 1.2%, indicating that the quality of sequencing was reliable.

### The gut microbiome of obese children, overweight children and controls

**Fig. 1:**
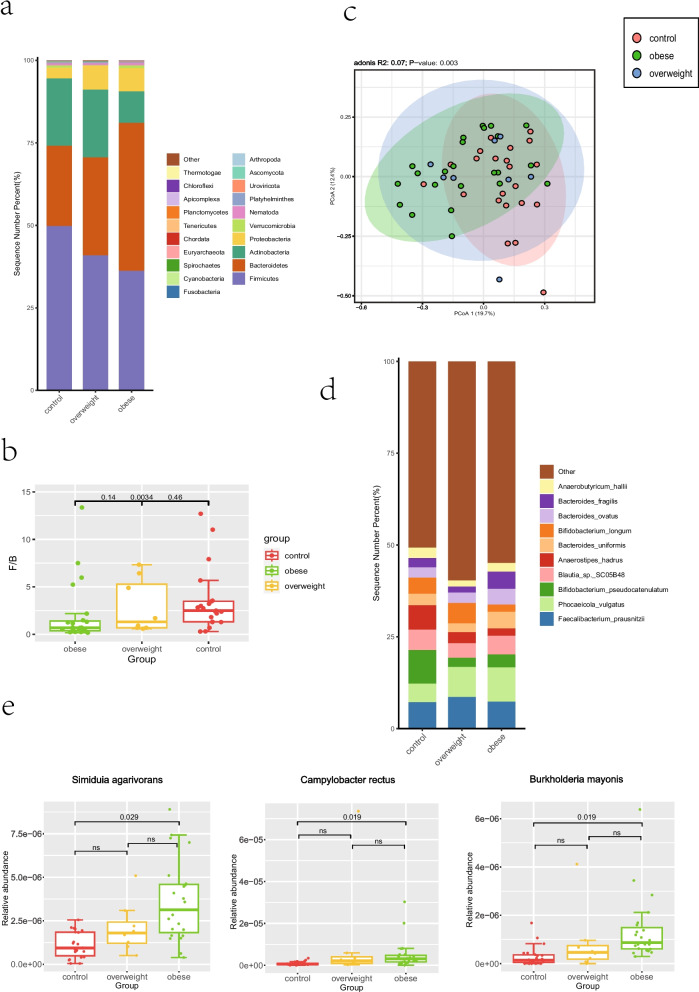
The gut microbiota composition of obese children, overweight children and the control group. **a** The gut microbial structures of controls, overweight and obese children at the phylum level. **b** The differences among the intestinal F/B ratio of controls, overweight and obese children (Mann–Whitney U test; *p* values correspond to tests between groups in the following order: obese vs overweight, obese vs control, and overweight vs control). **c** Principal coordinate analysis of the gut microbiota at the species level in controls, overweight and obese children (PERMANOVA test of Bray–Curtis dissimilarity). **d** The gut microbial structures in controls, overweight and obese children at the species level. **e** The relative abundance of differentially abundant species in the guts of obese children, overweight children and controls (q values are shown above the corresponding boxplots)

We compared the gut microbiota of obese children, overweight children and controls to characterize differences in gut microbial composition. The gut microbiota of controls, overweight children and obese children was dominated by Firmicutes (42.5% ± 20.1%), Bacteroidetes (34.1% ± 22.72%), Actinobacteria (5.7% ± 15.8%) and Proteobacteria (5.5% ± 8.2%) at the phylum level (Fig. 1a). Furthermore, we calculated the Firmicutes/Bacteroidetes (F/B) ratio of obese children (1.98 ± 3.18), overweight children (2.91 ± 2.84) and controls (3.46 ± 3.22) and found that the controls had a significantly higher F/B ratio than obese children (Fig. 1b; Mann–Whitney U test; *p* < 0.01). Since F/B ratio can also be influenced by age, we calculated the F/B ratio in different age groups and found that age had no significant effect on F/B ratio (Mann–Whitney U test; *p* > 0.05; Additional file 3).

We performed alpha‐diversity analysis by calculating the Shannon, Simpson, Chao1, ACE, observed_species and Good’s coverage index at the species level. We detected no significant differences in the gut microbial richness or diversity among obese children, overweight children and controls (Fig. S1m; Mann–Whitney U test; *p* > 0.05). PCoA plot based on the species level relative abundance profile showed that PCoA1 explained 19.7% of the variability and PCoA2 explained 12.4% of the variability (Fig. 1c). The PERMANOVA (Permutational multivariate analysis of variance) test of the Bray–Curtis dissimilarity showed a significant difference in the samples between obese children and controls (PERMANOVA test of Bray–Curtis dissimilarity; *p* = 0.001), while there was no significant difference in the samples between obese children and overweight children (PERMANOVA test of Bray–Curtis dissimilarity; *p* = 0.381), nor between overweight children and controls (PERMANOVA test of Bray–Curtis dissimilarity; *p* = 0.162).

The top five genera in relative abundance in the gut microbiota of controls were *Bifidobacterium* (17.8% ± 17.1%), *Bacteroides* (15.2% ± 11.0%), *Faecalibacterium* (7.2% ± 6.3%), *Blautia* (7.1% ± 4.9%) and *Anaerostipes* (6.7% ± 7.4%) (Fig. S2a). There were five top genera in the guts of obese children, namely, *Bacteroides* (24.7% ± 15.0%), *Phocaeicola* (12.7% ± 12.4%), *Bifidobacterium* (7.8% ± 8.9%), *Faecalibacterium* (7.1% ± 4.2%) and *Alistipes* (5.8% ± 9.5%), while *Bifidobacterium* (19.4% ± 26.8%), *Bacteroides* (14.8% ± 11.8%), *Phocaeicola* (10.4% ± 11.1%), *Faecalibacterium* (8.4% ± 6.5%) and *Blautia* (4.6% ± 2.4%) were the top five genera in the guts of overweight children (Fig. S2a). At species level, *Bifidobacterium pseudocatenulatum* (9.2% ± 9.4%), *Faecalibacterium prausnitzii* (7.2% ± 6.1%), *Anaerostipes hadrus* (6.7% ± 7.8%), *Blautia sp. SC05B48* (5.5% ± 4.5%) and *Phocaeicola vulgatus* (5.0% ± 6.9%) were the top five species in relative abundance in the control group, while *Bifidobacterium breve* (9.3% ± 25.9%), *Faecalibacterium prausnitzii* (8.6% ± 6.5%), *Phocaeicola vulgatus* (8.2% ± 10.3%), *Bifidobacterium longum* (5.6% ± 10.0%) and *Blautia_sp._SC05B48* (4.0% ± 2.2%) were the top five species in the overweight group (Fig. 1d). In the obese group, *Phocaeicola vulgatus* (9.3% ± 10.9%), *Faecalibacterium prausnitzii* (7.3% ± 4.5%), *Blautia sp. SC05B48* (5.0% ± 5.3%), *Bacteroides fragilis* (4.7% ± 7.5%) and *Bacteroides uniformis* (4.5% ± 3.7%) were the five most abundant species (Fig. 1d).

We also compared the relative abundance of gut microbes at the species level among obese children, overweight children and controls. Only *Simiduia agarivorans*, *Campylobacter rectus* and *Burkholderia mayonis* were significantly more abundant in the guts of obese children than in controls (Fig. 1e; Mann–Whitney U test; FDR < 0.05). There was also a gradient in relative abundance of these three species from the guts of obese children to controls (Fig. 1e).

**Fig. 2:**
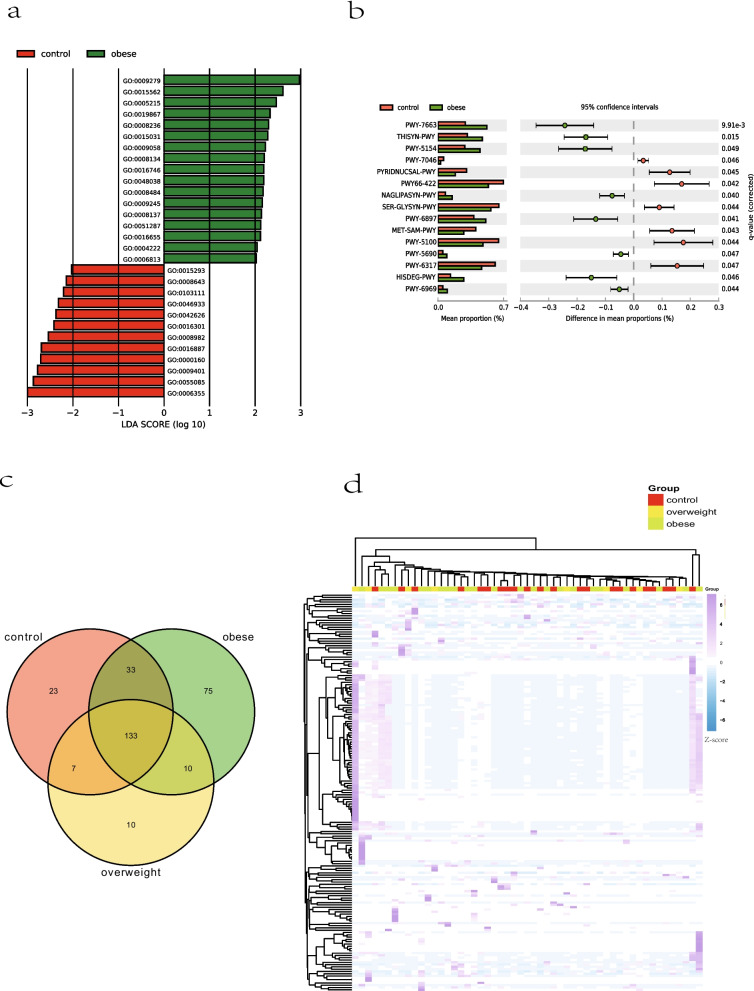
Functional differences in the gut microbiome among obese children, overweight children and controls. **a** GO enrichment analysis between controls and obese children (Kruskal–Wallis test; FDR < 0.05, LDA > 2). **b** Microbial metabolic pathway enrichment analysis in obese children and controls (Welch’s t-test; FDR < 0.05). **c** The number of ARGs in obese children, overweight children and controls. **d** Heatmap analysis of ARGs in obese children, overweight children and controls

Functional annotation of genes of the gut microbiota can provide further insight into the functional differences in the gut microbiome among obese children, overweight children and controls. For Gene Ontology (GO) enrichment analysis, GO terms associated with transporter (GO:0015562 for efflux transmembrane transporter activity; GO:0005215 for transporter activity; GO:0015031 for protein transport) and outer membrane (GO:0009279 for cell outer membrane; GO:0019867 for outer membrane) were more abundant in gut microbial genes in obese children than in controls (Fig. 2a; Kruskal–Wallis test; FDR < 0.05; LDA > 2). The genes associated with serine-type peptidase activity (GO:0008236), sulfuric ester hydrolase activity (GO:0008484), and lipid A biosynthetic process (GO:0009245) were also more abundant in the gut microbiome of obese children, while the genes associated with kinase activity (GO:0016301) was more abundant in the gut microbiome of control children (Fig. 2a; Kruskal–Wallis test; FDR < 0.05; LDA > 2). Microbial metabolic pathway enrichment analysis indicated that L-arginine biosynthesis (PWY-5154), L-histidine degradation I (HISDEG-PWY), TCA cycle II (PWY-5690) and TCA cycle V (2-oxoglutarate synthase) (PWY-6969) pathways were enriched in the gut microbiota of obese children (Fig. 2b; Welch’s t-test; FDR < 0.05). Meanwhile, D-galactose degradation V (Leloir pathway) (PWY66-422) and L-serine and glycine biosynthesis I (SER-GLYSYN-PWY) pathways were enriched in the gut microbiota of control group (Fig. 2b; Welch’s t-test; FDR < 0.05). Analysis of antibiotic resistance genes (ARGs) showed that the gut microbiota of obese children had more ARGs than that of overweight children and controls (Fig. 2c). Obese group contained totally 251 ARGs, while overweight group and control group contained 160 and 196 ARGs, respectively. Obese, overweight and control group shared 133 ARGs. From the heatmap (Fig. 2d), it can be seen that there was no obvious clustering of groups based on the abundance of ARGs among the gut microbiota of obese and overweight children and controls. There were no significantly differentially abundant ARGs, KEGG K numbers or CAZymes after FDR correction among obese children, overweight children and control children (Additional file 3).

### The gut microbiome of obese children in China and Mexico

Mexico is a country with a high prevalence of obesity (a combined prevalence of obesity and overweight of 33.2% in children; 2016) [11], and their eating habits might account for it [11, 12]. We compared the differences in the gut microbiome between obese Chinese children and obese Mexican children to explore how living habits effect their gut microbiome. The data of Mexican children was downloaded from a public database (see Methods). The average age of these obese Mexican children was 120.0 ± 2.4 (month), and their average BMI was 24.6 ± 0.8 (kg/m^2^). The basic characteristics of obese Mexican children were shown in Table 2.

**Table 2:** The basic characteristics of obese Chinese children and obese Mexican children

	China (Obese)	Mexico (Obese)	*p value*
Number of children, n	23	10	-
Male, n (%)	9 (39.1)	5 (50)	> 0.05^a^
Months of age (m) ($\overline{X }$±SD)	108.9 ± 36.9	120 ± 2.9	> 0.05^b^
BMI (kg.m^−2^) ($\overline{X }$±SD)	23.8 ± 3.3	24.6 ± 0.8	> 0.05^b^

^a^Refer to the Fisher's exact test

^b^Refer to the Mann–Whitney U test

**Fig. 3:**
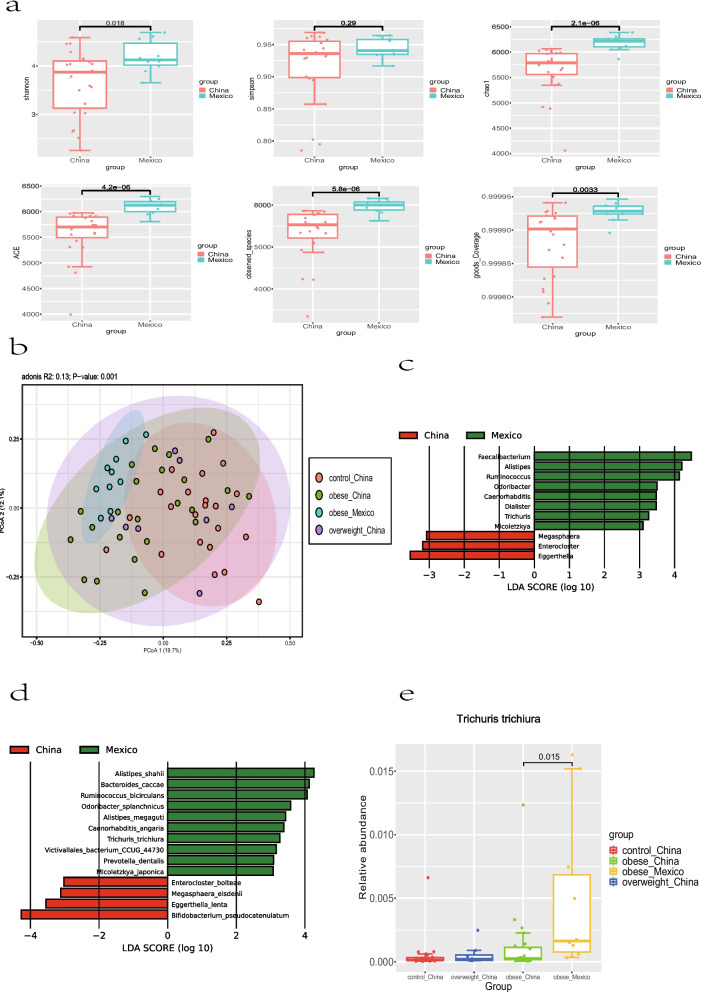
The gut microbiota composition of obese children in China and Mexico. **a** Alpha‐diversity analysis of the gut microbiota at the species level (Mann–Whitney U test; *p* values are shown above the corresponding boxplots). **b** Principal coordinate analysis of the gut microbiota at the species level (PERMANOVA test of Bray–Curtis dissimilarity). **c** LEfSe analysis of gut microbiota in obese children in China and Mexico at the genus level (Kruskal–Wallis test; FDR < 0.05, LDA > 3). **d** LEfSe analysis of gut microbiota in obese children in China and Mexico at the species level (Kruskal–Wallis test; FDR < 0.05, LDA > 3). **e** The relative abundance of *Trichuris trichiura* in Chinese children and Mexican children (q value that we are focusing on is shown above the corresponding boxplots)

We performed alpha diversity analysis (Fig. 3a) and found that the Shannon index of obese Mexican children was significantly higher than that of obese Chinese children (Mann–Whitney U test; *p* < 0.05). Obese Mexican children also had a higher Chao1, ACE and number of observed species than obese Chinese children (Fig. 3a; Mann–Whitney U test; *p* < 0.001). The Good’s coverage of both Mexican and obese Chinese children was over 99.975% (Fig. 3a). Therefore, the guts of obese Mexican children had higher diversity and richness than those of obese Chinese children. The PERMANOVA test of Bray–Curtis dissimilarity at the species level showed a significant difference in the samples between obese Chinese children and obese Mexican children (Fig. 3b; PERMANOVA test of Bray–Curtis dissimilarity; *p* = 0.002). Also, there was a significant difference in the samples between obese Mexican children and Chinese controls (PERMANOVA test of Bray–Curtis dissimilarity; *p* = 0.001), as well as obese Mexican children and overweight Chinese children (PERMANOVA test of Bray–Curtis dissimilarity; *p* = 0.001).

The guts of obese children from Mexico were also dominated by Bacteroidetes (46.7% ± 11.7%), Firmicutes (40.0% ± 14.2%), Proteobacteria (4.4% ± 3.2%) and Actinobacteria (3.1% ± 1.0%) at the phylum level (Fig. S2b). The relative abundance of Actinobacteria in the gut of obese Chinese children was significantly higher than that in Mexican children, while Nematoda, Platyhelminthes and Lentisphaerae were significantly more abundant in the gut of obese Mexican children (Kruskal–Wallis test; FDR < 0.05; LDA > 3). There was no significant difference in the F/B ratio between obese children in China (1.98 ± 3.18) and Mexico (0.98 ± 0.57) (Fig. S2c; Mann–Whitney U test; *p* > 0.05).

The top five genera in the guts of obese Mexican children were *Bacteroides* (24.8% ± 8.6%), *Faecalibacterium* (12.7% ± 6.9%), *Phocaeicola* (11.2% ± 6.5%), *Alistipes* (8.8% ± 5.2%) and *Prevotella* (3.6% ± 5.8%) (Fig. S2d). *Eggerthella*, *Enterocloster* and *Megasphaera* were significantly less abundant in the guts of obese Mexican children compared with obese Chinese children, while *Faecalibacterium*, *Alistipes*, *Ruminococcus*, *Odoribacter*, *Caenorhabditis*, *Dialister*, *Trichuris* and *Micoletzkya* were significantly more abundant in the guts of obese Mexican children (Fig. 3c; Kruskal–Wallis test; FDR < 0.05; LDA > 3). *Odoribacter splanchnicus*, *Ruminococcus bicirculans*, *Bacteroides caccae*, *Alistipes shahii* and *Trichuris trichiura* were significantly more abundant in Mexican children, while *Bifidobacterium pseudocatenulatum*, *Enterocloster bolteae*, *Megasphaera elsdenii* and *Eggerthella lenta* were significantly more abundant in Chinese children (Fig. 3d; Kruskal–Wallis test; FDR < 0.05; LDA > 3). Also, there was a gradient in relative abundance of *Trichuris trichiura* from the guts of Chinese controls to obese Mexican (Fig. 3e).

## Discussion

In this study, we observed a distinct difference in the gut microbiome between obese children and controls and further determined that the differential gut microbiota composition, microbial gene families and metabolic pathways might be related to the development of obesity. The intestinal F/B ratio was once thought to reflect dysbiosis of the gut microbiota, such as obesity [13]. However, in recent years, the effect of the F/B ratio on obesity has produced conflicting results in different studies. A study in Nanjing City of China showed that there was no significant difference in the F/B ratio between obese children and controls [14], while another study in Guangzhou City of China showed that obese children had a higher F/B ratio than the control group [15]. Moreover, in a meta-analysis, obese patients had a lower F/B ratio than controls [16]. In our study, the F/B ratio of obese children was significantly lower than that in the control group. Based on the above conflicting results, we speculated that the F/B ratio may be related to geography or individual lifestyle rather than BMI. The relatively small sample size of the studies on obese children also served as a limitation. Therefore, more research is required to define whether the F/B ratio can be used to identify obesity in children.

In addition, there might be gut inflammation in obese children*. Campylobacter rectus*, a mobile gram-negative rod, is an oral pathogen commonly found in oral cavity and is associated with periodontitis [17], but it has also been reported to cause thoracic empyema [18, 19] and destructive osteomyelitis of the sternum [20]. An obese female who developed severe extensor tenosynovitis from *Campylobacter rectus* after a dog bite [21]. These cases confirmed the potential for invasive infections with oral pathogens, especially in people with poor immunity and oral hygiene. In our study, compared with the control group, the *Campylobacter rectus* increased significantly in the gut of obese children, so it is speculated that obese children might have poor oral hygiene and a potential risk of infection in the gut. Moreover, serine-type peptidase activity, sulfuric ester hydrolase activity and lipid A biosynthetic process were more enriched in gut microbiome of obese children compared with controls. Serine-type peptidase activity, catalysis of the hydrolysis of peptide bonds in a polypeptide chain by a catalytic mechanism that involves a catalytic triad consisting of a serine nucleophile, was enriched in fecal microbiome of patients with ulcerative colitis [22], which implied that serine-type peptidase was associated with inflammation. The mucus secreted by the colon can form a barrier between the microbes and the intestinal epithelium, while some bacteria are able to use mucus glycoproteins (the main components of mucus) as a source of nutrients through sulfuric ester hydrolase, and thus may cause inflammatory bowel diseases [23]. Lipid A is the main immunostimulatory part of the LPS (lipopolysaccharide, a characteristic component of the cell wall of Gram-negative bacteria), and is classed as a virulence factor due to its high endotoxicity [24]. The enrichment of serine-type peptidase activity, sulfuric ester hydrolase activity and lipid A biosynthetic process in the gut microbiome of obese children implied that obese children might have inflammation in their intestines. Obese patients are more likely to be infected [25] and may therefore receive more antibiotics, so the number of ARGs in the gut microbiota of obese children was much higher than that of control children. A healthy diet can lower ARGs in obese children [26].

Also, there might be changes of gut microbial metabolism in obese children. GO terms related to outer membrane and transporter activity (cell outer membrane, efflux transmembrane transporter activity, transporter activity, outer membrane and protein transport) were more abundant in gut microbial genes in obese children than in controls, which implied an increased metabolism of gut microbes. Also, TCA cycle II and TCA cycle V pathways were enriched in the gut microbiota of obese children, which might indicate that there was a more active gut microbiome metabolism of aerobic respiration in obese children. L-serine and glycine biosynthesis was reduced in the gut microbiota of obese children, which might also induce metabolic disorders in obese children since glycine has beneficial effects on metabolic disorders associated with obesity, type 2 diabetes (T2DM), and non-alcoholic fatty liver disease (NAFLDs) [27]. Additionally, there were some beneficial pathways in the obese group. We found that genes involve in L-arginine biosynthesis were more abundant in the gut microbiota of obese children. Obesity-associated bacteria produce L-arginine, which upregulates NKG2D expression in γδ T cells and fights against HSV-2 virus infection through "pseudonormoxia" [28]. Histidine supplementation could suppress inflammation and oxidative stress [29], and genes involved in histidine degradation were more abundant in the gut microbiota of obese children.

Moreover, there was a gradient in relative abundance of differentially abundant species (*Simiduia agarivorans*, *Campylobacter rectus* and *Burkholderia mayonis*) and F/B ratio from the guts of obese children to controls. The PCoA plot and the PERMANOVA test of Bray–Curtis dissimilarity of obese group, overweight group and control group also implied that the gut status of overweight children may be in a transition state between obese children and control children.

We also compared the gut microbiota of obese children in China and Mexico and found that the gut microbiota of obese Mexican children was different from that of obese Chinese children. First, both of the Chinese children and obese Mexican children may have intestinal parasitic infections, since *Trichuris trichiura* was detected in their guts. *Trichuris trichiura* can cause trichuriasis [30], which is prevalent in warm, moist, tropical and subtropical regions of the world [31]. Sichuan province is in the subtropical zone with a humid climate and the Greater Mexico City is in the tropical zone, which might account for the occurrence of *Trichuris trichiura*. There was more *Trichuris trichiura* in the guts of obese Mexican children, which might be due to poor sanitation and lack of clean water in some households [32, 33]. Changes in the gut microbiome might also be related to the eating habits of obese children. Studies have shown that an animal-based diet can significantly increase fecal deoxycholic acid (DCA, a secondary bile acid) concentrations and lead to an increase in the abundance of bile-tolerant microorganisms such as *Alistipes* and *Bacteroides* [34]. The increased relative abundance of bile-tolerant microorganisms, such as *Alistipes*, *Odoribacter splanchnicus* [35] and *Bacteroides caccae* in the guts of obese Mexican children compared with obese Chinese children in our study implied a preference for an animal-based diet in Mexico. Many members of *Bifidobacterium* are health-promoting species, which can increase the levels of glucagon-like peptide-1 in both the gut and plasma, reduce visceral fat accumulation [36] and promote a healthier microvillus environment [37]. *Bifidobacterium pseudocatenulatum* can improve inflammatory status in children with obesity [38]. *Bacteroides caccae* is an intestinal opportunistic pathogenic bacteria, which can invade the mucosa of the intestine and cause various abdominal suppurative infections [39]. The increased abundance of *Bacteroides caccae* and the reduced abundance of *Bifidobacterium pseudocatenulatum* might mean gut inflammation might be even worse in Mexican children. *Megasphaera* was significantly more abundant in the gut of obese Chinese children compared with that of obese Mexican children, and it was reported that *Megasphaera* was enriched in the gut of Asians [40], which is consistent with our research. Since our study only analyzed the gut metagenome of obese children, we can only get a genetic profile of the gut microbes rather than the genes or gene expression in their hosts. Therefore, the genome, transcriptome and metabolome of obese children can be combined for subsequent analysis, so as to have a more complete understanding of the causes of childhood obesity. The limitation of this study also included the small sample size of overweight children with only 8 participants. A larger sample size might yield more accurate results. Different DNA extraction methods were applied by our research teams and Mexican research teams, which can also lead to the differences in composition of gut microbiome.

## Conclusions

In conclusion, our research revealed structural characteristics of the gut microbiome in obese children, overweight children and controls. Based on gut microbes, we found that obese Chinese children might suffer from intestinal inflammation since *Campylobacter rectus* and genes associated with lipid A biosynthetic process were significantly more abundant in the gut of obese children compared with controls. In addition, there might be changes of gut microbial metabolism in obese children. In terms of gut microbiome composition, overweight children might belong to a transition state between obese and control children. We also found that the gut microbiota of obese Mexican children had higher diversity and richness than those of obese Chinese children, and obese Mexican children had more abundant parasites. Our findings might shed light on the relationship between obesity and the gut microbiome.

## Methods

### Study design and subjects

Some of the participants were recruited from an embedded case‒control study of the Childhood Obesity Health Management Platform in West China Second Hospital of Sichuan University in Chengdu, China. A total of 53 children (23 males/30 females, 4 ~ 15 years old) were finally enrolled in the study at the Pediatric Department from August 2021 to April 2022. The weight and height were accurately measured by trained researchers following strict protocols, and the children’s body mass index (BMI) was calculated by weight/height^2^ (kg.m^−2^). Childhood overweight/obesity screening was based on the guidelines for the prevention and control of childhood obesity in China in 2021 [41]. Children aged 4 ~ 6 years were confirmed as overweight with a BMI-for-age greater than 1 SD and confirmed as obese with a BMI-for-age greater than 2 SD above the World Health Organization (WHO) Growth Reference median (2007). Children aged 6 ~ 18 years were confirmed as overweight or obese during screening according to the sex and age-specific BMI reference for school-aged children. Finally, we included 23 obese children, 8 overweight children and 22 normal-weight controls in the study. The study was approved by the Medical Ethics Committee of West China Second Hospital of Sichuan University (NO. 2020, 092).

### Sample collection, sequencing and metagenomic analysis

Before collection, the methods and notes were explained by the researchers. The children's feces were collected only after their parents signed an informed consent to participate in the study. Feces were collected using a sterile kit and frozen at − 80 °C immediately until analysis.

Total DNA was extracted by using the Magnetic Soil And Stool DNA Kit (Tiangen Biotech Co., Ltd., China). All the samples were sequenced using an Illumina NovaSeq 6000 (Novogene Co., Ltd. China) with a paired-end sequencing length of 150 bp. The adapters and low-quality reads were filtered by Trimmomatic (v.0.39) [42], while potential human sequences were removed by Bowtie2 (v2.4.5) [43] based on the NCBI reference genome (hg38).

The taxonomic labels of metagenomic sequences were assigned using kraken2 (v2.1.2) [44] with the option “–use-mpa-style” based on the databases (20200624) of “archaea”, "bacteria", “viral”, “fungi” and “protozoa”. The abundances of taxa were normalized by relative abundance. All taxa were retained for subsequent analysis. MEGAHIT (v1.2.9) [45] was used to assemble the metagenome with the option “–min-contig-len 300”. The non-redundant gene set was constructed using CD-HIT (v4.8.1) [46] with the option “-c 0.95 -aS 0.90.” The quantification of these non-redundant genes in each sample was performed using Salmon (v0.13.1) [47]. The genes were translated into proteins by Prodigal (V2.6.3) [48].

Microbial gene families and metabolic pathways were assessed using HUMAnN3 (v3.0.1) [49] based on the UniRef90 EC filtered database (uniref90_v269_201901) [50], which can map to the Kyoto Encyclopedia of Genes and Genomes (KEGG) [51], Gene ontology [52] and MetaCyc [53] databases, and were normalized by CPM (count per million). The microbial amino acid sequences were aligned to the Carbohydrate-Active enZYmes (CAZy) database (CAZyDB.09242021) [54] via dbCAN2 [55]. Antibiotic resistance genes (ARGs) were quantified using RGI (v5.2.1) with the comprehensive antibiotic resistance database (CARD 3.2.2) [56].

The stacked column chart and heatmap were completed using Wekemo BioinCloud. Alpha-diversity analysis with six indexes (Shannon, Simpson, Chao1, ACE, observed_species and goods_coverage) was performed by R package ‘vegan’ (2.6–4) with Mann–Whitney U test. The version of R is 4.3.1. Mann–Whitney U test was also used to test the F/B ratio between two groups. Differentially abundant taxa (comparison between Mexican and Chinese) and GO terms were identified using LEfSe (v1.1.2), which used Kruskal–Wallis test and LDA score to screen for biomarkers. Since LEfSe only outputs uncorrected *p* values for features that it finds significant, we reset the parameter of run_lefse.py script to output all *p* values. Then all the *p* values were multiple testing corrected using Benjamini–Hochberg method. These corrected *p* values were substituted for the uncorrected *p* values for further analysis. PCoA was based on the Bray‒Curtis metric, and a PERMANOVA test on each PCoA was performed by using the adonis function of R to ensure significant separation of different groups. The R script for PCoA was provided in Additional file 4. Differentially abundant pathways was screened by STAMP (v2.1.3) based on Welch’s t-test (FDR < 0.05; Benjamini–Hochberg method). Differentially abundant species (comparison among Chinese children), ARGs, KEGG K numbers and CAZy enzymes were screened by Mann–Whitney U test (Benjamini–Hochberg method). The ARGs heatmap normalized the data using Z-score.

### Collection of public data

We downloaded the fecal metagenome data of 10 obese Mexican children from a public database [57]. These metagenomes were also filtered by using Trimmomatic (v.0.39) [42] and Bowtie2 (v2.4.5) [43] to remove low-quality reads and host sequences. The classification labels of these metagenomic sequences were assigned by using kraken2 (v2.1.2) [44], and the abundance of taxa was standardized by relative abundance.

### Supplementary Information

**Additional file 1. **

**Additional file 2. **

**Additional file 3. **

**Additional file 4. **

**Additional file 5. **

**Additional file 6. **

## Data Availability

The datasets generated during the current study are available in the CNGB Sequence Archive (CNSA) of China National GeneBank DataBase (CNGBdb) with accession number CNP0004872 (https://db.cngb.org/). The supplementary figures are presented in Additional file 2. The statistical outputs are presented in Additional file 3 and Additional file 5. The feature abundance tables are presented in Additional file 6.

## Abbreviations

PERMANOVA: Permutational multivariate analysis of variance
PCoA: Principal coordinates analysis
LEfSe: Linear discriminant analysis Effect Size
F/B: Firmicutes/Bacteroidetes
ARGs: Antibiotic resistance genes
GO: Gene Ontology
KEGG: Kyoto Encyclopedia of Genes and Genomes
