# Supplementary material for: Metagenomic analysis reveals distinct changes in the gut microbiome of obese Chinese children
Source: BMC Genomics. 2023 Nov 29;24:721. doi: 10.1186/s12864-023-09805-4 (PMC10685578; doi:10.1186/s12864-023-09805-4)
Supplement: Supplementary file 2 — Additional file 2. [file 12864_2023_9805_MOESM2_ESM.pdf]

Figure S1

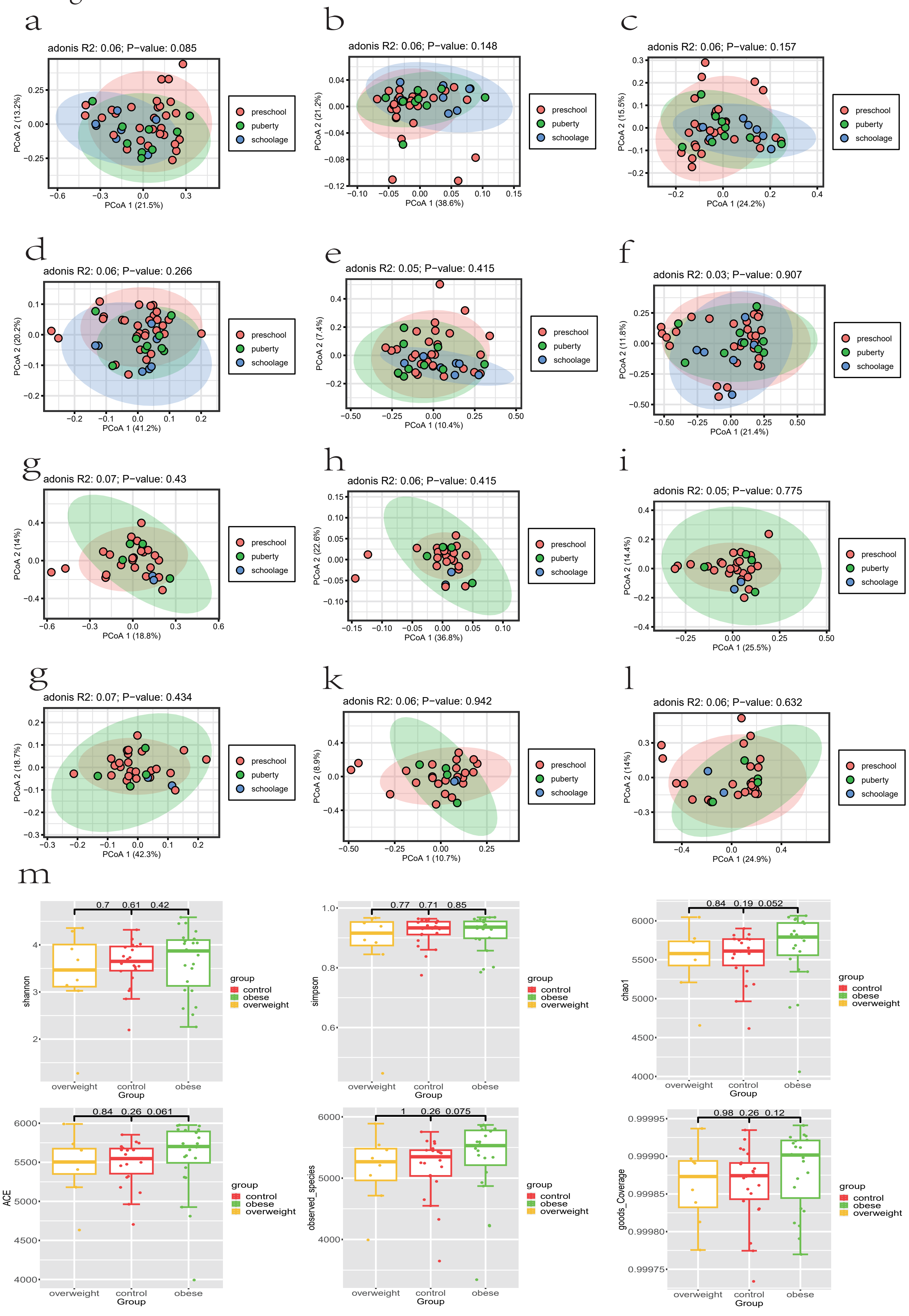
a
adonis R2: 0.06; P−value: 0.085
PCoA 1 (21.5%)
PCoA 2 (13.2%)
preschool
puberty
schoolage
b
adonis R2: 0.06; P−value: 0.148
PCoA 1 (38.6%)
PCoA 2 (21.2%)
c
adonis R2: 0.06; P−value: 0.157
PCoA 1 (24.2%)
PCoA 2 (15.5%)
d
adonis R2: 0.06; P−value: 0.266
PCoA 1 (41.2%)
PCoA 2 (20.2%)
e
adonis R2: 0.05; P−value: 0.415
PCoA 1 (10.4%)
PCoA 2 (7.4%)
f
adonis R2: 0.03; P−value: 0.907
PCoA 1 (21.4%)
PCoA 2 (11.8%)
g
adonis R2: 0.07; P−value: 0.43
PCoA 1 (18.8%)
PCoA 2 (14%)
h
adonis R2: 0.06; P−value: 0.415
PCoA 1 (36.8%)
PCoA 2 (22.6%)
i
adonis R2: 0.05; P−value: 0.775
PCoA 1 (25.5%)
PCoA 2 (14.4%)
g
adonis R2: 0.07; P−value: 0.434
PCoA 1 (42.3%)
PCoA 2 (18.7%)
k
adonis R2: 0.06; P−value: 0.942
PCoA 1 (10.7%)
PCoA 2 (8.9%)
l
adonis R2: 0.06; P−value: 0.632
PCoA 1 (24.9%)
PCoA 2 (14%)
m
0.7 0.61 0.42
shannon
0.77 0.71 0.85
simpson
0.84 0.19 0.052
chao1
0.84 0.26 0.061
ACE
1 0.26 0.075
observed_species
0.98 0.26 0.12
goods_Coverage
overweight
control
obese
Group
group
control
obese
overweight

Fig. S1

PCoA plot based on PERMANOVA test of Bray–Curtis distance of a) species, b) GO, c) KEGG, d) metabolic pathways, e) CAZy enzymes and f) ARGs of gut microbiota of Chinese obese children and control with different ages (4~6 years for preschool; 7~9 years for school age; 10~15 years for puberty).

PCoA plot based on Bray–Curtis distance of g) species, h) GO, i) KEGG, j) metabolic pathways, k) CAZy enzymes and l) ARGs of gut microbiota of Chinese overweight children and control with different ages (4~6 years for preschool; 7~9 years for school age; 10~15 years for puberty).

m) Alpha - diversity analysis of the gut microbiota of obese children, overweight children and controls at the species level (Mann-Whitney U test; p values correspond to tests between groups in the following order: overweight vs control, obese vs overweight, and obese vs control).

Figure S2

a

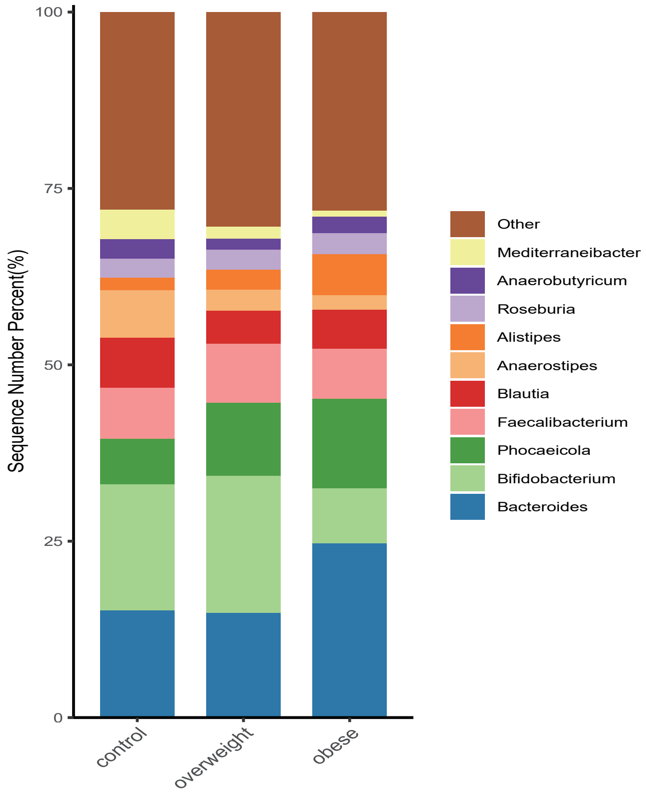

b

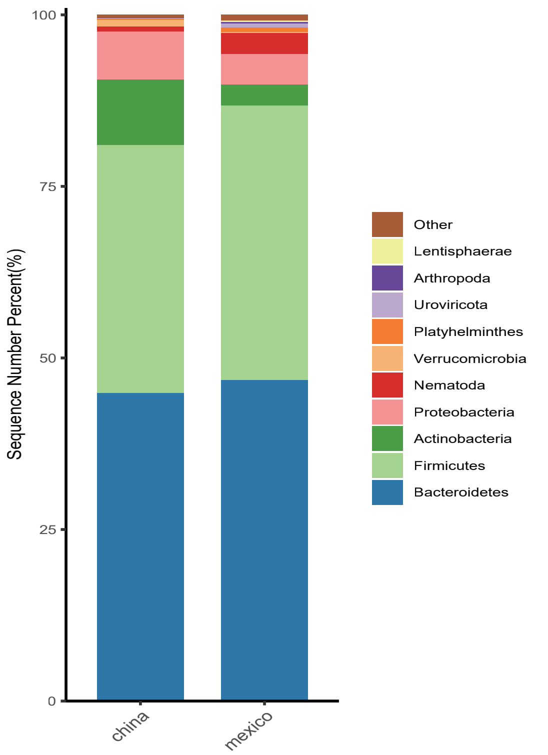

c

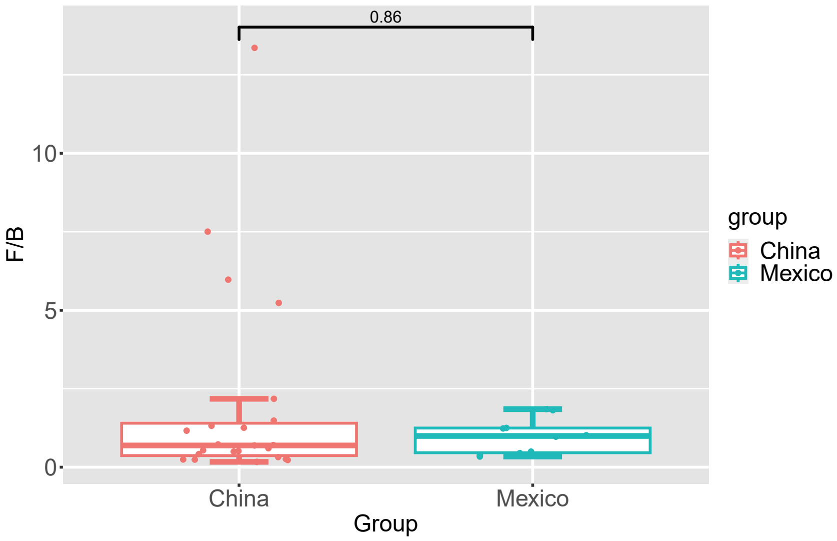

d

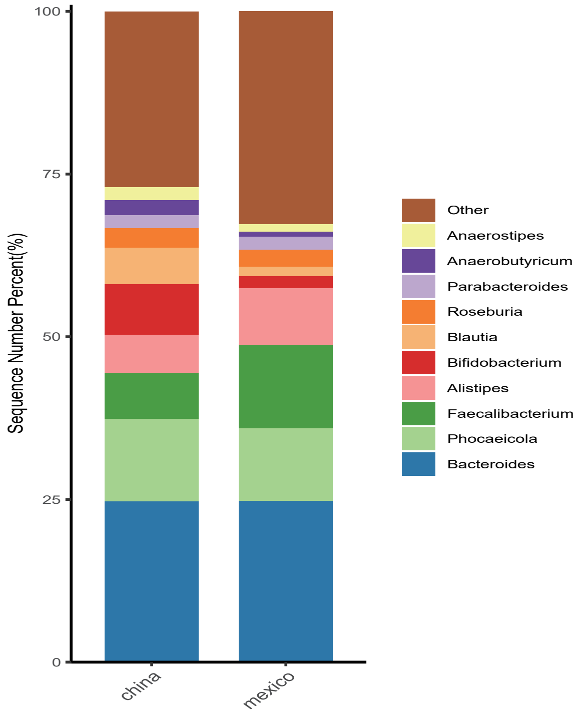

Fig. S2 a) The gut microbial structures of controls, obese children and overweight children at the genus level. b) The gut microbial structures of obese children in China and Mexico at the phylum level. c) The differences between the intestinal F/B ratio of obese children in China and Mexico (Mann-Whitney U test; p value is shown above the boxplots). d) The gut microbial structures of obese children in China and Mexico at the genus level.
