# Supplementary material for: Metagenomic analysis reveals distinct changes in the gut microbiome of obese Chinese children
Source: BMC Genomics. 2023 Nov 29;24:721. doi: 10.1186/s12864-023-09805-4 (PMC10685578; doi:10.1186/s12864-023-09805-4)
Supplement: Supplementary file 5 — Additional file 5. [file 12864_2023_9805_MOESM5_ESM.pdf]

Full statistical outputs for microbial metabolic pathways in different comparisions (Welch's t-test; Benjamini-Hochberg method).

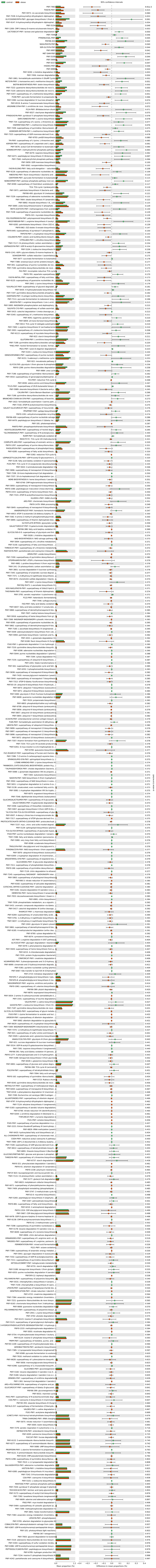
control
obese
95% confidence intervals
Mean proportion (%)
Difference in mean proportions (%)

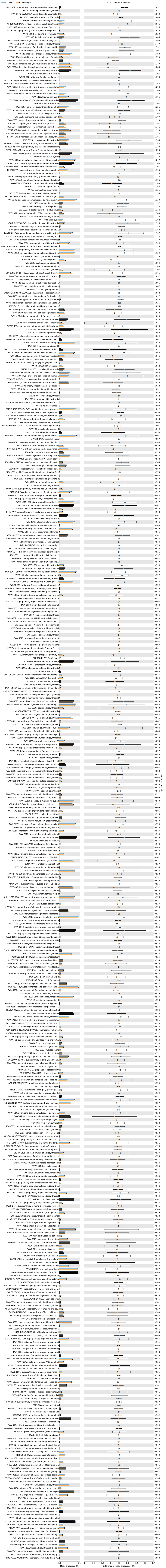
obese
overweight
95% confidence intervals

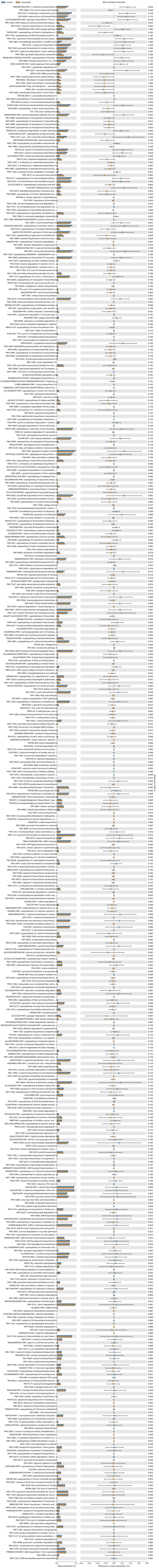
control
overweight
95% confidence intervals
Mean proportion (%)
Difference in mean proportions (%)
